# Preparation of 3DOM ZrTiO_4_ Support, W_x_CeMnO_δ_/3DOM ZrTiO_4_ Catalysts, and Their Catalytic Performance for the Simultaneous Removal of Soot and NO_x_

**DOI:** 10.3389/fchem.2022.880884

**Published:** 2022-05-04

**Authors:** Ruidan Wang, Chengming Zhong, Dong Li, Xuehua Yu, Zhen Zhao, Zbigniew Sojka, Andrzej Kotarba, Yuechang Wei, Jian Liu

**Affiliations:** ^1^ State Key Laboratory of Heavy Oil Processing, China University of Petroleum, Beijing, China; ^2^ Institute of Catalysis for Energy and Environment, College of Chemistry and Chemical Engineering, Shenyang Normal University, Shenyang, China; ^3^ Faculty of Chemistry, Jagiellonian University, Kraków, Poland

**Keywords:** three-dimensional ordered macroporous structure, soot particulates, nitrogen oxides, simultaneous removal, catalysts

## Abstract

As an efficient and durable engine, a diesel engine has a broad application. However, soot particles (PM) and nitrogen oxides (NO_x_) coming from diesel engines are the main causes of air pollution, so it is necessary to design and prepare an effective catalyst for the simultaneous elimination of PM and NO_x_. In this work, a novel 3DOM ZrTiO_4_ support and a series of W_x_CeMnO_δ_/3DOM ZrTiO_4_ catalysts (where x indicates the wt% of W) were designed and fabricated by the colloidal crystal template technique. Among the as-prepared catalysts, the W_1_CeMnO_δ_/3DOM ZrTiO_4_ catalyst exhibits the highest NO conversion rate (52%) at the temperature of maximum CO_2_ concentration (474°C) and achieves 90% NO conversion in the temperature range of 250–396°C. The excellent catalytic performance is associated with the macroporous structure, abundant oxygen vacancies, sufficient acid sites, and the synergistic effect among the active components. The possible reaction mechanisms of W_x_CeMnO_δ_/3DOM ZrTiO_4_ catalysts were also discussed based on the characterization results.

## 1 Introduction

Diesel engines are ideal for heavy-duty vehicles, with high durability, lower cost, and longevity (Wei et al., 2011; Chen et al., 2016; Dai et al., 2016; Cheng et al., 2017a). However, a lot of soot particles (PM) and nitrogen oxides (NO_x_) are simultaneously generated during diesel combustion (Epling et al., 2004; Liu and Gao, 2011). PM and NO_x_ are the main causes of urban haze weather, which can lead to serious environmental pollution and health problems (Zhu et al., 2008; Li et al., 2012; Shen et al., 2013; Choi and Lee, 2014). As more and more stringent emission standards are set by governments, there is a growing interest in developing technologies which enable the reduction of such emissions.

At present, after-treatment technologies are widely used to eliminate soot particulates and NO_x_ (Gogos et al., 2014; Urán et al., 2019); that is, a catalyzed diesel particulate filter (CDPF) is applied to eliminate soot particulates (Wei et al., 2011; Yu et al., 2017), and the selective catalytic reduction (SCR) technology or the nitrogen oxide storage reduction (NSR) technology is simultaneously matched for the NO_x_ removal (Li et al., 2015; Li et al., 2016). However, traditionally, the after-treatment technique possesses some inherited flaws, such as big system volume, large mass, and high cost. In view of this, the idea of the concurrent catalytic elimination of soot particles and NO_x_ in a single trap is attractive and has been initially projected by Yoshida et al. (1989). This approach has attracted great interest from researchers, as it reduces the pressure and shrinks the volume and mass of the system. However, a few shortcomings are still there and are yet to be overcome. For example, the PM combustion process is a characteristic gas–solid–solid catalytic reaction; in general, the diameter of PM is larger than the pore diameter of conventional catalysts, so the soot particles cannot be efficiently transported through the pores of catalytic materials and the active sites cannot be fully used either. In addition, NO_x_ conversion takes place in a lean-burn condition, but the excessive O_2_ and insufficient reductant will result in a low NO_x_ conversion.

Many catalysts have been developed for the simultaneous elimination of soot particulates and NO_x_, such as noble metals (Serhan et al., 2019), metal oxides (Bueno-López et al., 2016; Cheng et al., 2017b), perovskite oxides (Liu et al., 2008; Li et al., 2010; Wang et al., 2010), zeolites (Fritz and Pitchon, 1997), and spinel phases (Lin et al., 2009). Among these materials, metal oxides have shown excellent performances (Li et al., 2009; Shen et al., 2013). Mn-based catalysts have been proven to be exceptional in PM combustion and SCR reactions (Chen et al., 2019; Yu D. et al., 2021; Peng et al., 2021). As a promoter, even active catalysts, namely, CeO_2_-containing materials, are applied widely because of their excellent ability for oxygen storage and the aptitude to shift between Ce^4+^ and Ce ^3+^ under a stipulated redox environment (Jin et al., 2014). Specifically, tungsten species have been reported to have the function to advance the catalytic availability of active sites, oxygen-related vacancy, and acid-introduced sites (Xiong et al., 2017). In addition, TiO_2_ is always busy as support for SCR catalysts (Wu et al., 2011), and by the way, adding additional elements may increase the surface area and thermal stability, and enhance the surface acidity.

Herein, a new type of ZrTiO_4_ support with a three-dimensional ordered macroporous (3DOM) structure was synthesized by the colloidal crystal templating (CCT) technique, which is rarely reported in the previous literature. Meanwhile, three cheap active elements of W, Ce, and Mn were added to the 3DOM ZrTiO_4_ support by a simple and convenient method to simultaneously remove nitrogen oxides and soot particles from diesel exhausts. The as-prepared catalysts have a three-dimensional ordered, inter-connected macroporous structure, which can effectively improve the contact performance between reactants and catalytic active sites, and facilitate the effective transmission of particulate reactants. NH_3_ as a reducing gas was also introduced in order to improve the reduction of NO_x_.

## 2 Experimental

### 2.1 Material Preparation

#### 2.1.1 Synthesis of 3DOM ZrTiO_4_ Support

The 3DOM ZrTiO_4_ support was fabricated by the colloidal crystal templating (CCT) technique, and the template was prepared by using polymethyl methacrylate (PMMA) spheres (Xu et al., 2011). In a typical process, C_16_H_36_O_4_Ti and ZrOCl_2_·8H_2_O (at a molar ratio of 1:1) were taken in the mixed solution of methanol and ethylene glycol (at a volume ratio of 3:7), and strongly stirred followed by the addition of the PMMA template. The mixture was maintained for 3 h until the PMMA templates had been fully impregnated. The excess precursor solution was separated by vacuum filtration. Subsequently, the final precipitate was maintained at 80°C for 12 h to dry. After that, the dried precipitate was calcined at 550°C for 4 h with a raising rate of 1°C/min to remove the template. Finally, 3DOM ZrTiO_4_ support was obtained.

#### 2.1.2 Synthesis of 3DOM ZrTiO_4_ Supported Catalysts

The 3DOM ZrTiO_4_ supported catalysts were synthesized by the incipient wetness impregnation method. (NH_4_)_6_H_2_W_12_O_40_·xH_2_O, Ce(NO_3_)_3_·6H_2_O, and Mn(NO_3_)_2_·4H_2_O with different mass ratios were dissolved in distilled water, and then the mixture was added dropwise into the 0.5 g ZrTiO4 support. The volume of the mixture should be equal to the pore volume of the 3DOM ZrTiO_4_ support. These precursors were dried at 80°C for 12 h, and the dried precipitate was calcined at 550°C for 4 h at a rate of 1°C/min. Finally, W_x_CeMnO_δ_/3DOM ZrTiO_4_ catalysts were obtained, where x means the mass percent of W to ZrTiO_4_ support. To obtain single metal oxide supported on 3DOM ZrTiO_4_ catalysts, (NH_4_)_6_H_2_W_12_O_40_·xH_2_O, Ce(NO_3_)_3_·6H_2_O, and Mn(NO_3_)_2_·4H_2_O were individually dissolved in distilled water, and they were added into the ZrTiO_4_ support dropwise. Next, the treated processes are similar to those of W_x_CeMnO_δ_/3DOM ZrTiO_4_ catalysts. Finally, Mn_2_O_3_/3DOM ZrTiO_4_, CeO_2_/3DOM ZrTiO_4_, and WO_3_/3DOM ZrTiO_4_ were obtained.

#### 2.1.3 Synthesis of W_1_CeMnO_δ_/3DOM TiO_2_ and W_1_CeMnO_δ_/3DOM ZrO_2_ Catalysts

For comparison, W_1_CeMnO_δ_/3DOM TiO_2_ and W_1_CeMnO_δ_/3DOM ZrO_2_ samples were also prepared by the incipient wetness impregnation method. A similar procedure for the preparation of 3DOM ZrTiO_4_ was applied for the preparation of 3DOM TiO_2_ and 3DOM ZrO_2_ supports. 3DOM TiO_2_ and 3DOM ZrO_2_ supports were impregnated by the same impregnation solution as that of the W_1_CeMnO_δ_/3DOM ZrTiO_4_ catalyst. The obtained precursor was dried at 80°C for 12 h, and after calculation at 550°C for 4 h, W_1_CeMnO_δ_/3DOM TiO_2_ and W_1_CeMnO_δ_/3DOM ZrO_2_ catalysts were obtained.

### 2.2 Material Characterization

The phase composition and crystal structure were evaluated by an X-ray diffractometer (Ultima IV, Rigaku) using Cu-K_α_ radiation as the source with a Ni filter. Nitrogen adsorption–desorption investigation was achieved with a Micromeritics TriStar II: 3020 analyzer to get the textural characteristics of catalysts. The morphology and microstructure of the catalysts were scrutinized by scanning electron microscopy (SEM, Zeiss sigma 500) and transmission electron microscopy (TEM, JEM-F200). Mn 2p, W 4f, Ce 3d, and O 1s binding energies were measured by X-ray photoelectron spectroscopy (XPS, PHI-1600 ESCA). H_2_-TPR measurements were conducted using AutoChemi II2920 equipment; 0.05 g sample was pretreated in N_2_ for 1 h at 300°C, followed by cooling down to 25°C. Thereafter, the flow gas was altered to 10-vol% H_2_/N_2_ with a maximum temperature of 900°C, and the heat treatment rate was kept at 10°C/min; hydrogen consumption was examined using a thermal conductivity detector (TCD). NH_3_-TPD was carried out on a conventional flow apparatus, and 0.05 g sample was heated at 600°C in N_2_ atmosphere for 1 h and saturated with 1% NH_3_ for 1 h. When the temperature cooled to room temperature, N_2_ was used to abolish the feebly attached NH_3_. At last, the sample was heated to 600°C at a rate of 10°C/min. *In situ* diffuse reflectance infrared Fourier transform (DRIFT) spectroscopy (Thermo Nicolet Is50 spectrometer) was used to investigate the SCR reaction mechanism of the W_1_CeMnO_δ_/3DOM ZrTiO_4_ catalyst. The catalyst was first purged with NH_3_ or NO + O_2_ at 200 and 300°C until adsorption was saturated. Then, the NH_3_ or NO + O_2_ were closed. After that, the reaction system was purged by N_2_, and then, the other corresponding reacted gases were introduced into the *in situ* reaction cell, and the FT-IR spectra were recorded at different times.

### 2.3 Catalytic Activity Tests

A fixed bed reactor was applied to evaluate the performance of the as-prepared catalysts for the simultaneous elimination of PM and NO_x_. The reaction gases comprised 1,000 ppm NH_3_, 1,000 ppm NO, and 5% O_2_; the balance gas was N_2_; and the total flow rate of the gases was 100 ml/min. The catalyst (100 mg) and PM (10 mg) were mixed together, and PM was simulated by Printex-U (Degussa). The reacted gas concentrations (including NH_3_, N_2_O, NO, NO_2_, CO, and CO_2_) were tested by the infrared spectrometer. An accurate and reliable quantitative method was established to measure the multiple gaseous components (Qin and Cadet, 1997; Valencia et al., 2009; Sinelli et al., 2010; Stec et al., 2011). The catalytic performance of the oxidation PM was evaluated by the value of T_m_, which was defined as the temperature for maximum CO_2_ concentration released. The NO reduction was defined by the highest conversion of NO to N_2_, and the conversion rate was calculated as follows (Zhang et al., 2011):

$$\mathrm{NO}\;\mathrm{Conversion}=\frac{{\left[\mathrm{NO}\right]}_{\mathrm{inlet}}-{\left[\mathrm{NO}\right]}_{\mathrm{outlet}}}{{\left[\mathrm{NO}\right]}_{\mathrm{inlet}}}\times 100\%,$$

in which [NO]_inlet_ and [NO]_outlet_, respectively, denote the inlet and outlet concentrations of NO under steady-state conditions.

## 3 Results and Discussion

### 3.1 Activity Tests of the Catalysts

**FIGURE 1:**
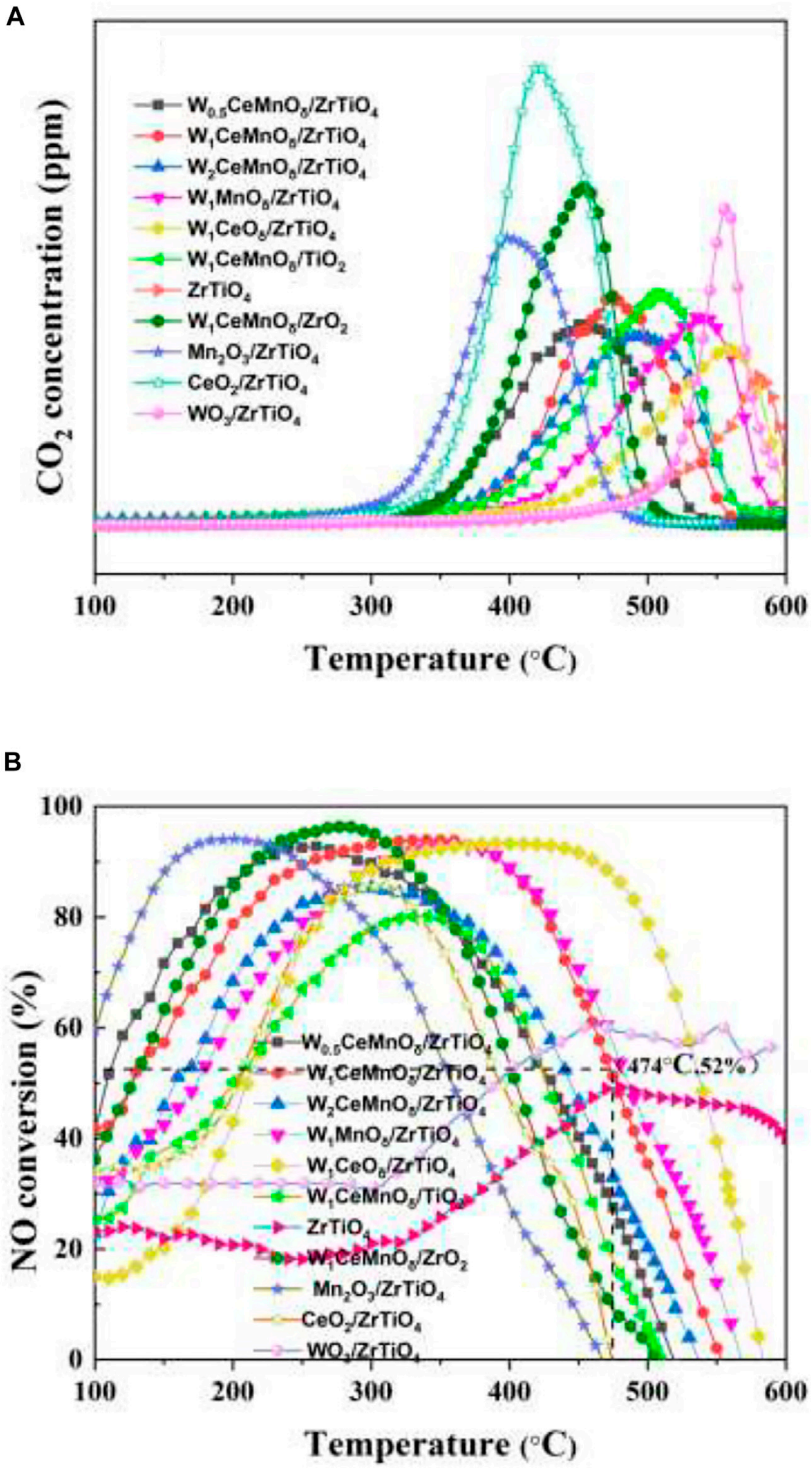
CO_2_ concentration cures **(A)** and NO conversion curves **(B)** for the simultaneous elimination of PM and NO_x_ over 3DOM catalysts.

The activities of W_x_CeMnO_δ_/3DOM ZrTiO_4_ catalysts for the simultaneous elimination of PM and NO_x_ were evaluated, and the results are shown in Figure 1 and Table 1. The lower T_m_ of PM oxidation means high catalytic efficiency, which is important for the design and preparation of catalysts. As shown in Figure 1A and Table 1, the T_m_ value of 3DOM ZrTiO_4_ support for the removal of PM is 581°C, indicating that the catalytic activity of the support is very weak. Compared with Mn_2_O_3_/3DOM ZrTiO_4_ and CeO_2_/3DOM ZrTiO_4_ catalysts, the WO_3_/3DOM ZrTiO_4_ catalyst has the highest T_m_ value of 559°C. When W is combined with Ce or Mn, the T_m_ of the bimetallic supported catalysts decreases. The T_m_ values of the W_1_MnO_δ_/3DOM ZrTiO_4_ and W_1_CeO_δ_/3DOM ZrTiO_4_ catalysts are 536 and 558°C, respectively. Interestingly, for the W_x_CeMnO_δ_/3DOM ZrTiO_4_ catalysts, the T_m_ values are all below 500°C. With increasing doping amounts of W, T_m_ is slightly raised. This indicates that W has a low activity for soot removal, and its activity can be greatly improved by combining with Ce and Mn. Compared with the W_1_CeMnO_δ_/3DOM TiO_2_ catalyst, the W_1_CeMnO_δ_/3DOM ZrO_2_ catalyst has a lower T_m_ value of 453°C, and the W_1_CeMnO_δ_/3DOM ZrTiO_4_ catalyst has the T_m_ value of 474°C, which indicates that the catalytic activity can be improved by adding Zr to Ti.

**TABLE 1:** Peak temperature of maximum CO_2_ concentration and 90% temperature window of NO conversion over 3DOM catalysts.

Catalysts	T_m_ ^a^/°C	T_NO_,_90_ ^b^/°C
ZrTiO_4_	581	
W_1_CeMnO_δ_/TiO_2_	504	
W_1_CeMnO_δ_/ZrO_2_	453	217–323
Mn_2_O_3_/ZrTiO_4_	400	157–245
CeO_2_/ZrTiO_4_	420	
WO_3_/ZrTiO_4_	559	
W_1_MnO_δ_/ZrTiO_4_	536	308–394
W_1_CeO_δ_/ZrTiO_4_	558	313–462
W_0.5_CeMnO_δ_/ZrTiO_4_	450	220–299
W_1_CeMnO_δ_/ZrTiO_4_	474	250–396
W_2_CeMnO_δ_/ZrTiO_4_	492	

a Peak temperature of maximum CO_2_ concentration.

b 90% temperature window of NO conversion.

The catalytic performance of NO reduction was evaluated by an operating temperature window (90% NO conversion). As shown in Figure 1B and Table 1, 3DOM ZrTiO_4_ support shows poor performance for NO conversion, and the conversion of over 50% can hardly be obtained on the ZrTiO_4_ support. Similarly, the WO_3_/ZrTiO_4_ catalyst also shows poor NO elimination performance. It is worth noting that the conversion window (90% NO conversion) of the Mn_2_O_3_/ZrTiO_4_ catalyst is only 88°C (157–245°C), and the CeO_2_/ZrTiO_4_ catalyst has no conversion window over 90%; when W is added to Mn and Ce, respectively, the conversion window of the W_1_MnO_δ_/ZrTiO_4_ catalyst has barely changed, but the conversion window of W_1_CeO_δ_/ZrTiO_4_ has been greatly improved; the W_1_CeMnO_δ_/ZrTiO_4_ catalyst not only exhibits the wide temperature window (250–396°C) but also has high NO conversion, which manifests that the interaction of Ce and W can widen the temperature window, and Mn has the effect of improving the NO conversion. The W_x_CeMnO_δ_/3DOM ZrTiO_4_ catalysts exhibit good NO conversion performance except for the W_2_CeMnO_δ_/3DOM ZrTiO_4_, indicating that excessive W doping is not beneficial for NO reduction. Compared with W_1_CeMnO_δ_/3DOM ZrTiO_4_, W_1_CeMnO_δ_/3DOM TiO_2_ and W_1_CeMnO_δ_/3DOM ZrO_2_ exhibit low catalytic activity. Therefore, the synergistic effect between Zr and Ti in ZrTiO_4_ support indeed has a positive effect to improve the catalytic performance. Based on the above analysis, W_1_CeMnO_δ_/3DOM ZrTiO_4_ catalyst not only exhibits the widest temperature window (250–396°C) at a lower temperature for 90% NO conversion but also has the highest NO conversion rate (52%) at the temperature of T_m_, which illustrates that the W_1_CeMnO_δ_/ZrTiO_4_ catalyst can be considered as one kind of talented catalysts for the simultaneous elimination of soot particles and nitrogen oxides.

### 3.2 XRD Analysis

**FIGURE 2:**
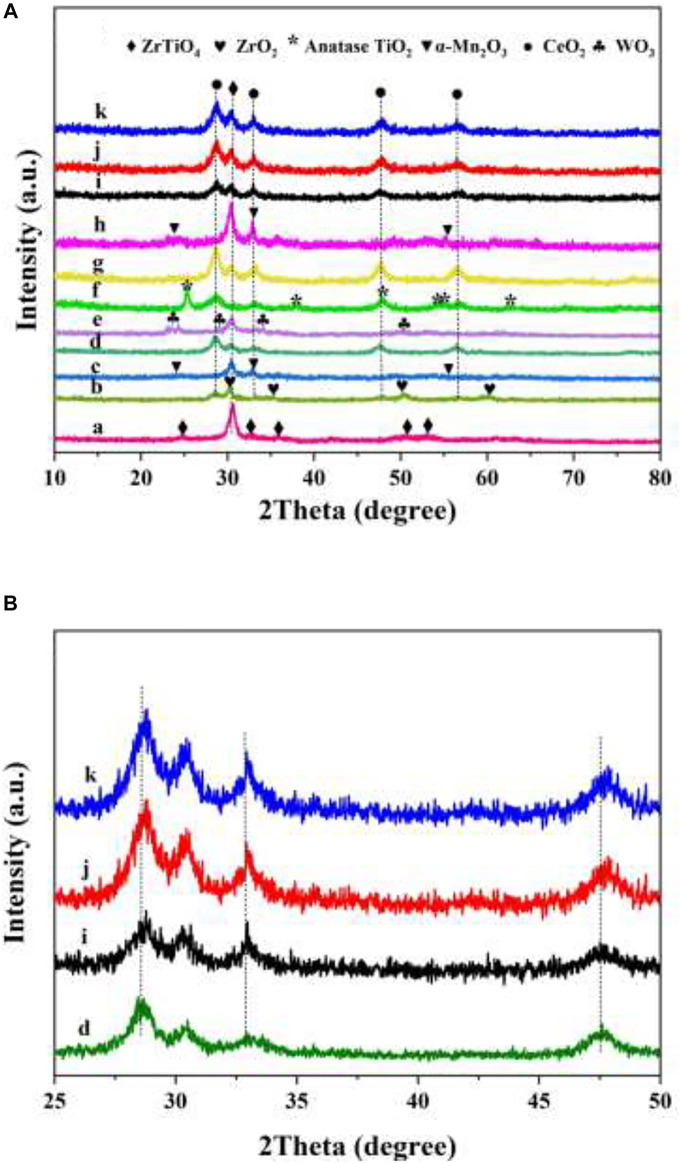
XRD patterns **(A)** 10–80°, **(B)** 25–50°of 3DOM materials. (a) ZrTiO_4_; (b) W_1_CeMnO_δ_/ZrO_2_; (c) Mn_2_O_3_/ZrTiO_4_; (d) CeO_2_/ZrTiO_4_; (e) WO_3_/ZrTiO_4_; (f)W_1_CeMnO_δ_/TiO_2_; (g) W_1_CeO_δ_/ZrTiO_4_; (h) W_1_MnO_δ_/ZrTiO_4_; (i) W_0.5_CeMnO_δ_/ZrTiO_4_; (j) W_1_CeMnO_δ_/ZrTiO_4_; (k) W_2_CeMnO_δ_/ZrTiO_4_.

The crystal structures of the as-prepared 3DOM ZrTiO_4_ support and it-supported catalysts were characterized by XRD measurements, and the results are exhibited in Figure 2. As shown in Figure 2, the main characteristic peaks at 2θ = 24.75°, 30.51°, 32.82°, 35.66°, 50.27°, and 52.98° can be assigned to the (110), (111), (020), (002), (202), and (221) crystal faces of ZrTiO_4_ support (JCPDS No. 80-1783) (Zhang Y. et al., 2015). The peaks at 2θ = 28.50°, 32.76°, 47.82°, and 56.51° of each sample can be assigned to the (111), (200), (220), and (311) crystal faces of cubic CeO_2_ (JCPDS PDF# 43-1002) (Lin et al., 2018). The characteristic peaks of manganese and tungsten oxide cannot be observed because the ionic radius of Mn and W are smaller than that of Ce. Therefore, Mn ions and W ions can easily enter the lattice of CeO_2_. As shown in Figure 2B, the diffraction peak of W_x_CeMnO_δ_/3DOM ZrTiO_4_ catalysts shifts to a higher angle with respect to the diffraction peak of the CeO_2_/3DOM ZrTiO_4_ catalyst (i.e., the peak at 2θ = 28.5° shifts to 28.7°), which confirms the above conclusion. The peaks at 2θ = 32.95°, 55.19°, and 23.13° can be assigned to the crystal faces (222), (440), and (211) of α-Mn_2_O_3_ for Mn_2_O_3_/3DOM ZrTiO_4_ and W_1_MnO_δ_/3DOM ZrTiO_4_ catalysts (JCPDS No.41-1442) (Saputra et al., 2014). For the W_1_CeMnO_δ_/3DOM TiO_2_ catalyst, the peaks at 2θ = 25.4°, 37.9°, 48.1°, 53.9°, 55.2°, and 62.8° belong to anatase TiO_2_, in which the crystal faces are (101), (004), (200), (105), (211), and (204), respectively (JCPDS No. 21-1272) (Jiao et al., 2017). For the W_1_CeMnO_δ_/3DOM ZrO_2_ catalyst, the peaks at 2θ = 30.3°, 35.3°, 50.4°, and 60.2° belong to the characteristic peak of ZrO_2_ (JCPDS No. 50-1089). For the WO_3_/3DOM ZrTiO_4_ catalyst, the peaks at 2θ = 23.2°, 23.7°, 24.4°, 28.8°, 34.0°, and 50.2° belong to the characteristic peak of WO_3_ (JCPDS No. 20-1323).

### 3.3 SEM, TEM, and EDS Mapping

**FIGURE 3:**
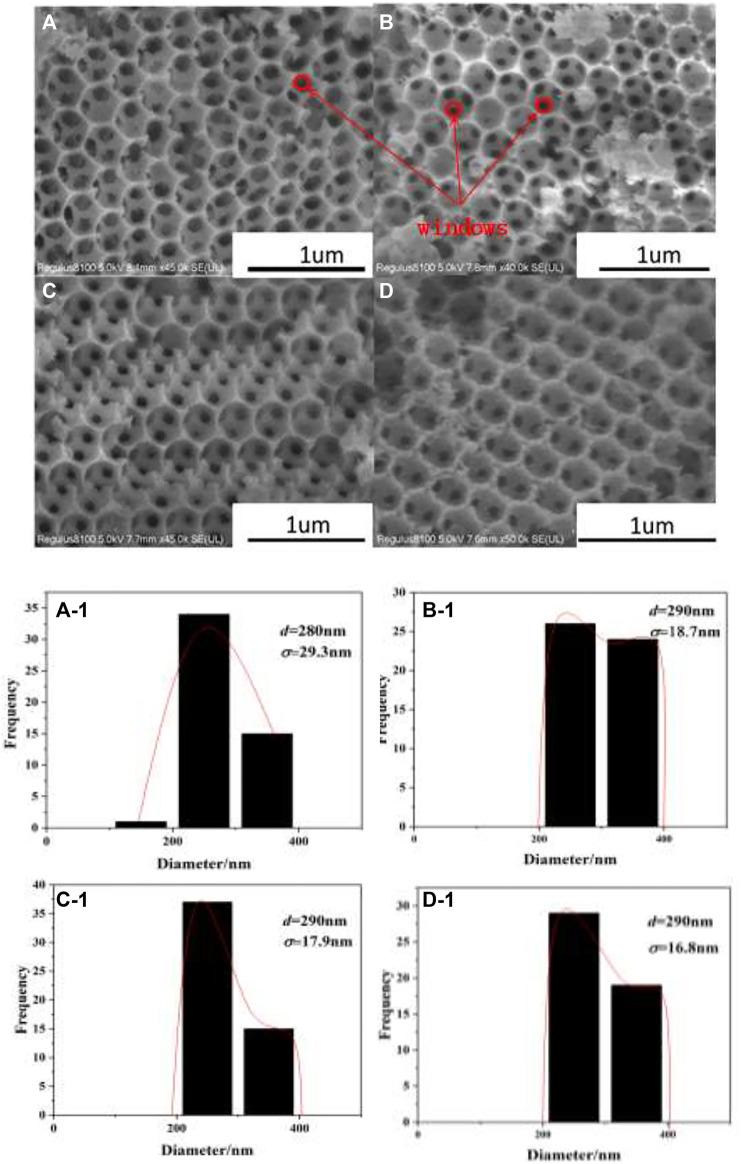
SEM images of 3DOM catalysts and histograms of macropores’ diameters. **(A)** W_1_CeO_δ_/ZrTiO_4_; **(B)** W_1_MnO_δ_/ZrTiO_4_; **(C)** W_1_CeMnO_δ_/ZrTiO_4_; **(D)** W_1_CeMnO_δ_/TiO_2_.

The SEM images, as shown in Figure 3, demonstrate that all catalysts have highly ordered macropores. From the distributions of macropores’ diameters for the as-prepared catalysts in Figures 3A-1–D-1, it can be seen that the average diameters of macropores are about 290 ± 20 nm. The diameters of macropores are lower than the PMMA diameter (400 nm), which is related to the shrinkage of polymer templates at high calcination temperature (Cheng et al., 2017a). The skeleton around macropores is constructed by uniform periodically arranged windows (marked with red circles in Figure 3), which form the layers through close linkage between the opening windows. Meanwhile, the highly ordered macropores for the as-prepared catalysts indicate the loading process of metal oxides does not destroy 3DOM structures.

**FIGURE 4:**
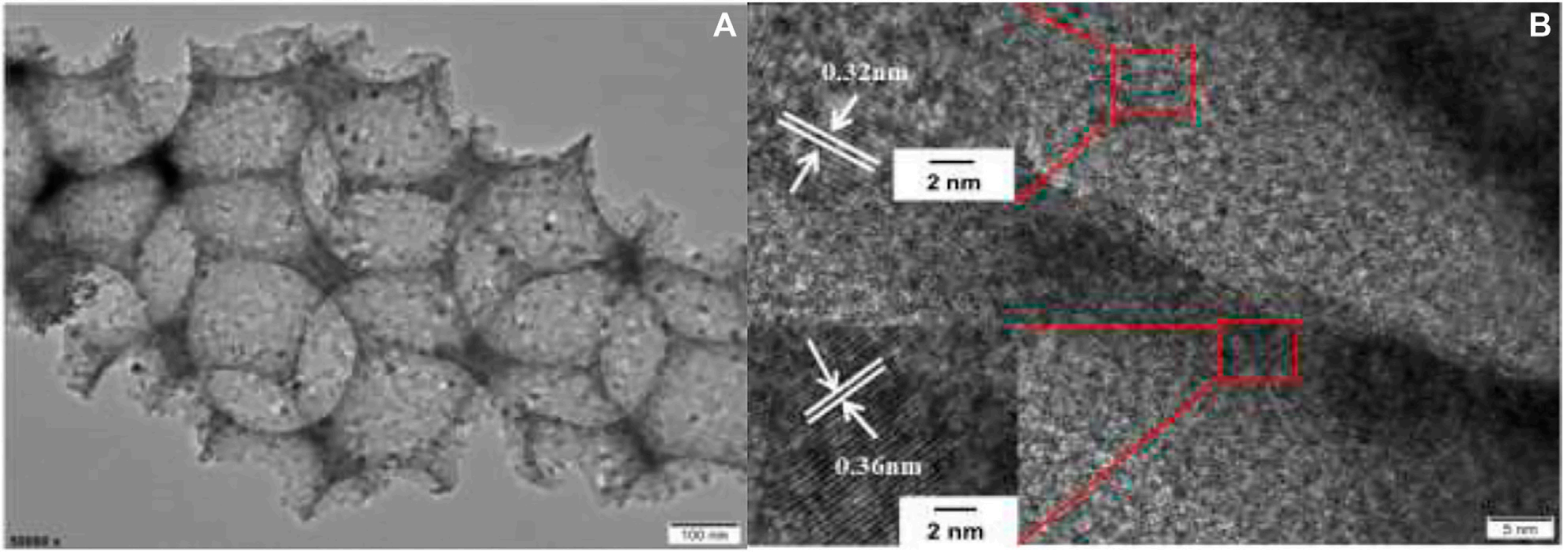
TEM **(A)** and HRTEM **(B)** images of W_1_CeMnO_δ_/3DOM ZrTiO_4_.

**FIGURE 5:**
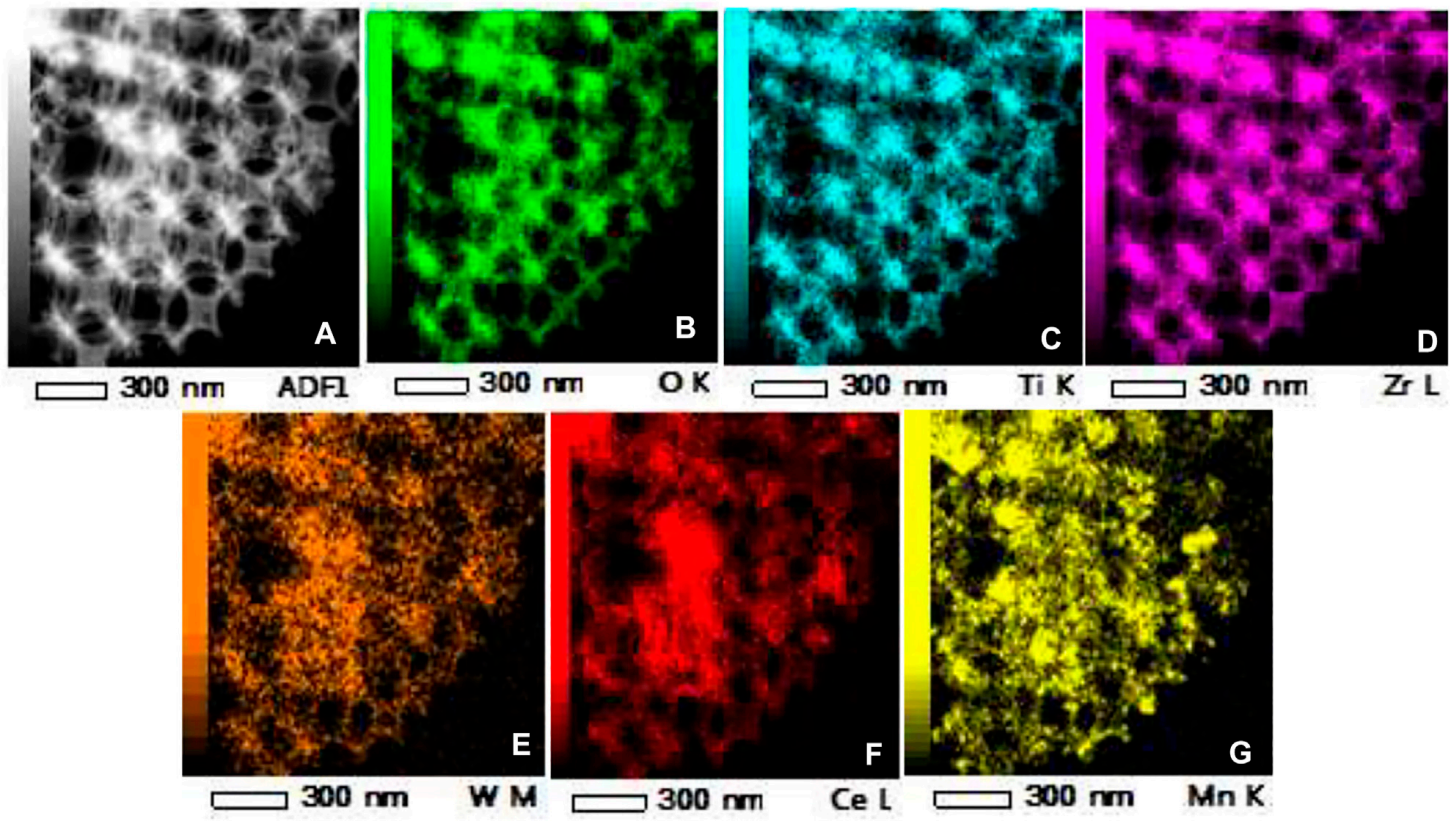
HAADF-STEM image and EDS elemental mappings of W_1_CeMnO_δ_/3DOM ZrTiO_4_. **(A)** STEM; **(B–G)** EDS mappings of O, Ti, Zr, W, Ce, and Mn.

TEM and HRTEM images of the W_1_CeMnO_δ_/3DOM ZrTiO_4_ catalyst are exhibited in Figure 4. Figure 4A shows that the W_1_CeMnO_δ_/3DOM ZrTiO_4_ catalyst has an ordered macroporous structure and the macropores are linked together by windows layer to layer. This well agrees with the SEM results. Furthermore, the surface of 3DOM ZrTiO_4_ is adhered by well-dispersed nanoparticles (NPs), and no large agglomerated particles can be obtained on the 3DOM skeleton, which indicates that metal oxides are evenly distributed on the surface of ZrTiO_4_. HRTEM image of W_1_CeMnO_δ_/3DOM ZrTiO_4_ is shown in Figure 4B; as observed from Figure 4B and the insert images of Figure 4B, the lattice fringes with a spacing of 0.32 nm are indexed as (111) planes of CeO_2_, and the second lattice fringes with a spacing of 0.36 nm correspond to (011) crystal plane of ZrTiO_4_. To study the distribution of W, Ce, Mn, Zr, Ti, and O elements in W_1_CeMnO_δ_/3DOM ZrTiO_4_, the HAADF-STEM images and EDS elemental mappings were obtained, and they are shown in Figure 5. From Figures 5B–D, the elements of O, Ti, and Zr cover the entire 3DOM skeleton because O, Ti, and Zr are the constituent elements of the support. From Figures 5E, F, it can be seen that the elements of W, Ce, and Mn are found throughout the surface of the catalyst, even inside the pores.

### 3.4 BET Study

**FIGURE 6:**
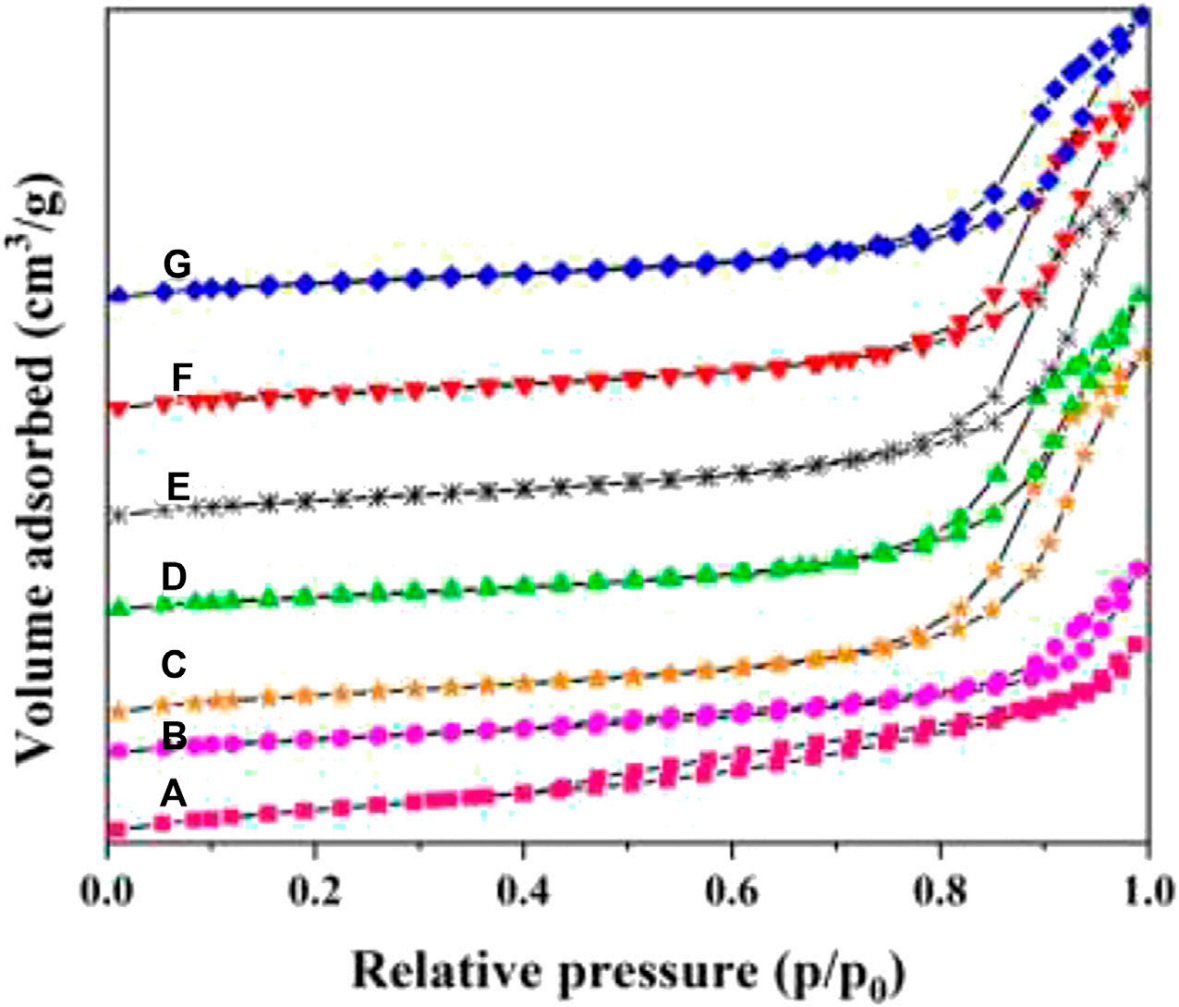
Nitrogen adsorption–desorption isotherms of 3DOM catalysts. **(A)** ZrTiO_4_; **(B)** W_1_MnO_δ_/ZrTiO_4_; **(C)** W_1_CeO_δ_/ZrTiO_4_; **(D)** W_1_CeMnO_δ_/TiO_2_; **(E)** W_0.5_CeMnO_δ_/ZrTiO_4_; **(F)** W_1_CeMnO_δ_/ZrTiO_4_; **(G)** W_2_CeMnO_δ_/ZrTiO_4_.

**FIGURE 7:**
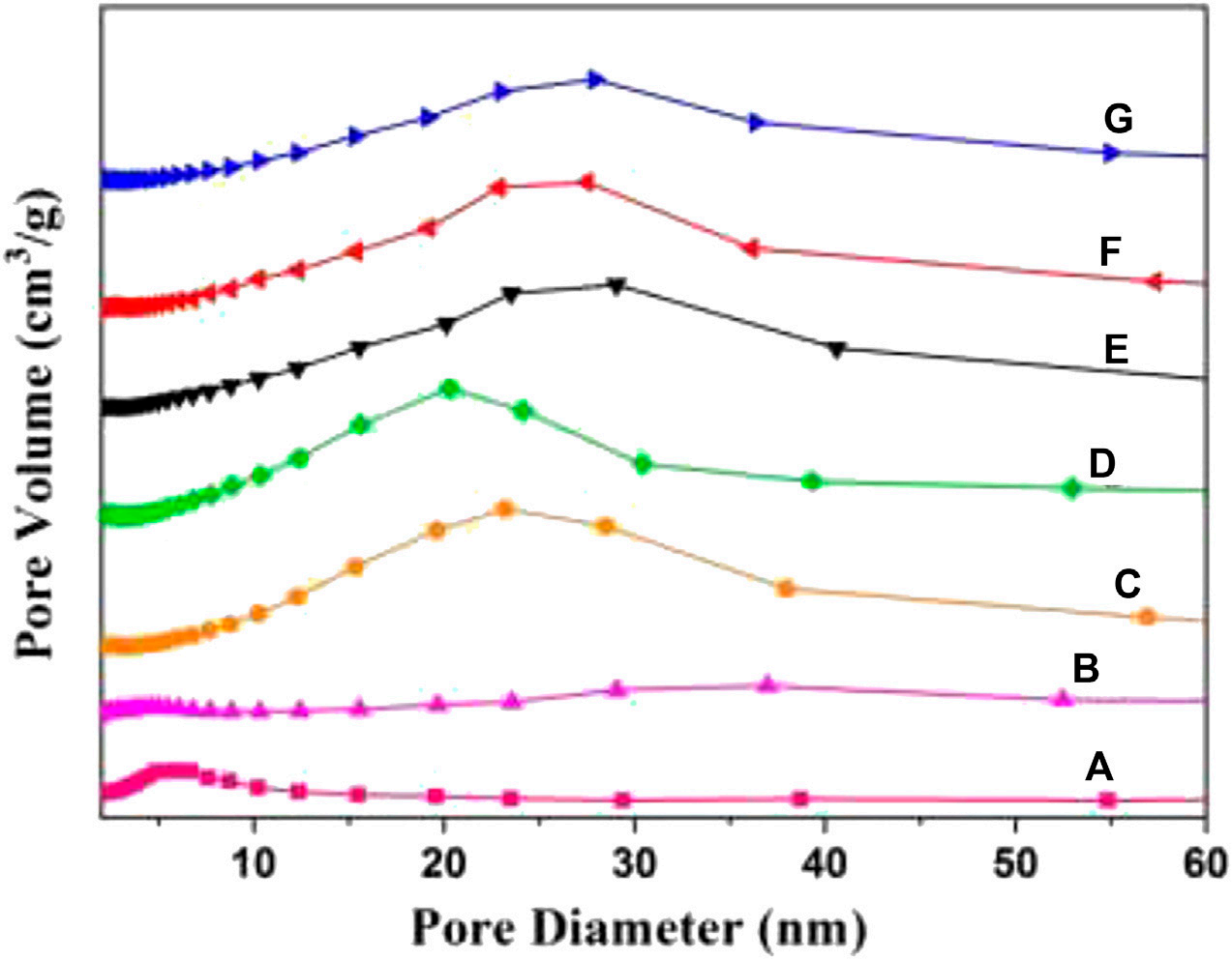
Mesoporous distribution curves of 3DOM catalysts. **(A)** ZrTiO_4_; **(B)** W_1_MnO_δ_/ZrTiO_4_; **(C)** W_1_CeO_δ_/ZrTiO_4_; **(D)** W_1_CeMnO_δ_/TiO_2_; **(E)** W_0.5_CeMnO_δ_/ZrTiO_4_; **(F)** W_1_CeMnO_δ_/ZrTiO_4_; **(G)** W_2_CeMnO_δ_/ZrTiO_4_.

The N_2_ adsorption–desorption isotherms and pore distribution curves of W_x_CeMnO_δ_/3DOM ZrTiO_4_ samples are presented in Figures 6, 7. The BET data of the as-prepared catalysts are summarized in Table 2. As shown in Figure 6, all the samples present typical II curves with a nearly linear relationship in the low-pressure vicinity. For 3DOM ZrTiO_4_ support, the H3 hysteresis loop increases slowly in the P/P_0_ range of 0.4–1.0, which may be due to the scraggly surface of the support. When the active components are loaded on the 3DOM ZrTiO_4_ support, the H3 hysteresis loop disappeared in the P/P_0_ range of 0.4–0.8 and the intensity in the P/P_0_ range of 0.8–1.0 increased sharply. This phenomenon may be due to the scraggly surface of the support being covered by the finely dispersed metal oxide, which makes the surface smooth (Xie et al., 2013).

**TABLE 2:** Textural properties of 3DOM ZrTiO_4_ and W_x_CeMnO_δ_/3DOM ZrTiO_4_ catalysts.

Catalysts	Surface area (m^2^/g)^a^	Total pore volume (cm^3^/g)^b^	Pore size (nm)^c^
ZrTiO_4_	54.0	0.098	6.2
W_1_MnO_δ_/ZrTiO_4_	37.0	0.095	8.9
W_1_CeO_δ_/ZrTiO_4_	48.1	0.177	13.7
W_1_CeMnO_δ_/TiO_2_	40.8	0.155	14.1
W_0.5_CeMnO_δ_/ZrTiO_4_	44.3	0.163	13.5
W_1_CeMnO_δ_/ZrTiO_4_	41.2	0.154	13.8
W_2_CeMnO_δ_/ZrTiO_4_	36.7	0.138	13.7

a Calculated by the BET method.

b Calculated by BJH desorption cumulative volume of pores between 1.7 and 300 nm diameter.

c Calculated by BJH desorption average pore diameter.

As shown in Figure 7, the as-prepared catalysts exhibit an obvious mesoporous structure. For 3DOM ZrTiO_4_ support, the mesopores with a diameter of 2–5 nm belong to the surface gap of the ZrTiO_4_ skeleton, and the mesopores with a diameter of 20–35 nm are associated with the accumulation of metal oxide NPs. As shown in Table 2, 3DOM ZrTiO_4_ exhibits the biggest surface area of 54.0 m^2^g^−1^, which may belong to the scraggly surface of ZrTiO_4_. However, the surface area decreased when active components are loaded on the ZrTiO_4_ support, which may be due to the covering of finely dispersed metal oxide on the scraggly surface. In addition, the surface area of W_1_CeMnO_δ_/3DOM ZrTiO_4_ is larger than that of W_1_CeMn/3DOM TiO_2_ due to the addition of ZrO_2_ to TiO_2_, which is in accordance with other reports (Zhang et al., 2012; Zhang Y. et al., 2015). Compared with the W_1_MnO_δ_/3DOM ZrTiO_4_ catalyst, W_1_CeMnO_δ_/3DOM ZrTiO_4_ exhibits a larger surface area, which is attributed to the addition of Ce. The loaded catalysts display a higher total pore volume and average pore size, and this is related to the accumulation effect of active components.

### 3.5 XPS Analysis

**FIGURE 8:**
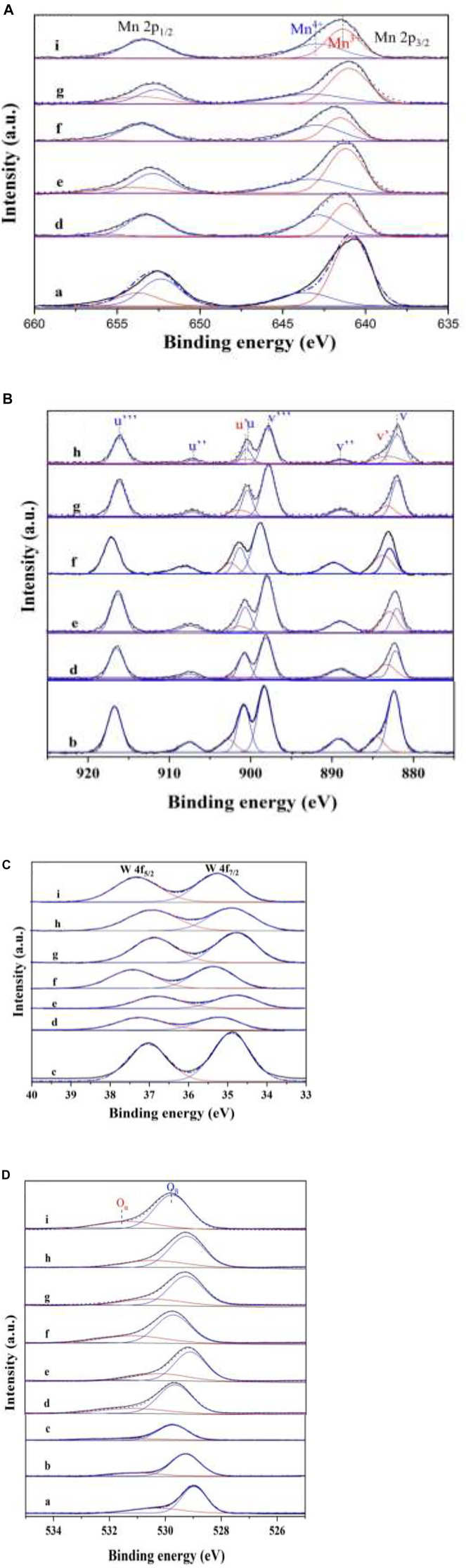
XPS spectra of Mn 2p **(A)**, Ce3d **(B)**, W4f **(C)**, and O 1s **(D)**. (a) Mn_2_O_3_; (b) CeO_2_; (c) WO_3_; (d) W_1_CeMnO_δ_/TiO_2_; (e) W_0.5_CeMnO_δ_/ZrTiO_4_; (f) W_1_CeMnO_δ_/ZrTiO_4_; (g) W_2_CeMnO_δ_/ZrTiO_4_; (h) W_1_CeO_δ_/ZrTiO_4_; (i) W_1_MnO_δ_/ZrTiO_4_.

To investigate the valence state of elements and surface composition of the as-prepared catalysts, XPS measurements were carried out, and the results are shown in Table 3 and Figure 8. The ratios of Mn^4+^/Mn^3+^, Ce^3+^/Ce^4+^, and (O^−^ + O_2_^−^)/O^2-^ of W_1_CeMnO_δ_/3DOM ZrTiO_4_ are comparatively higher than those of other catalysts, indicating that tungsten, ceria, manganese, and supports have strong interaction, which leads to the good catalytic performance of the W_1_CeMnO_δ_/3DOM ZrTiO_4_ catalyst.

**TABLE 3:** XPS result of W_x_CeMnO_δ_/3DOM ZrTiO_4_ catalysts.

	Mn^4+^/Mn^3+^ (%)	Ce^3+^/Ce^4+^ (%)	O_α_/O_β_ (%)
W_1_MnO_δ_/ZrTiO_4_	44.4		41.7
W_1_CeO_δ_/ZrTiO_4_		17.2	42.0
W_0.5_CeMnO_δ_/ZrTiO_4_	60.0	21.1	49.1
W_1_CeMnO_δ_/ZrTiO_4_	61.1	23.9	51.5
W_2_CeMnO_δ_/ZrTiO_4_	57.1	13.1	40.1
W_1_CeMnO_δ_/TiO_2_	49.6	13.6	40.6
Mn_2_O_3_	57.4		37.8
CeO_2_		12.9	21.3
WO_3_			27.1

Figure 8A shows the XPS profiles of Mn 2p for the as-prepared catalysts. The spectra of all the catalysts display two peaks. The peaks at 641.2 and 642.9 eV can be assigned to Mn^3+^ and Mn^4+^ (Li et al., 2016), respectively. It was well known that Mn^4+^ species are beneficial for low-temperature SCR (Yao et al., 2019; Li et al., 2021). As shown in Table 3, the ratio of Mn^4+^/Mn^3+^ of W_1_CeMnO_δ_/3DOM ZrTiO_4_ is higher than that of W_1_CeMnO_δ_/3DOM TiO_2_. In addition, the ratio of Mn^4+^/Mn^3+^ in the W_x_CeMnO_δ_/3DOM ZrTiO_4_ catalysts is higher than that of the WMnO_δ_/3DOM ZrTiO_4_ catalyst, which indicates the strong synergistic effects of Mn and Ce. Ce can accelerate the oxidation of Mn^3+^ to Mn^4+^; hence, more Mn^4+^can be produced.

Figure 8B shows the XPS profiles of Ce 3d. The peaks at about 900.5 and 883.5 eV denoted as u’ and v’ are the major peaks related to the 3d^10^4f^1^ state of Ce^3+^ ions, and the peaks at about 882.0, 888.8, 897.8, 900.4, 907.1, and 916.2 eV named as v, v’’, v’’’, u, u’’, and u’’’ are related to the 3d^10^4f^0^ state ascribed to Ce^4+^(Cheng et al., 2014). According to the peak area ratio of Ce^3+^ to Ce^4+^, the content of Ce^3+^ of monometallic oxide CeO_2_ is lowest in the as-prepared catalysts, indicating that the support and the doping of other metals can promote the production of more Ce^3+^. Meanwhile, the content of Ce^3+^ in W_1_CeMnO_δ_/3DOM ZrTiO_4_ is higher than that of W_1_CeMnO_δ_/3DOM TiO_2_, which indicates the synergistic effect of Zr and Ti. Similarly, the highest ratio of Ce^3+^/Ce^4+^ is also found in W_1_CeMnO_δ_/3DOM ZrTiO_4_, which indicates that the interaction between W and Ce will produce more Ce^3+^.

Figure 8C gives the W 4f_5/2_ and W 4f_7/2_ peaks at 36.6–37.6 and 34.7–35.7 assigned to W^6+^. As shown in Figure 8C, W_1_CeMnO_δ_/3DOM ZrTiO_4_ has higher binding energy than other catalysts. The higher binding energy generally represents the lower density of electron cloud, as W^6+^ has only four coordination bonds with surrounding O-atoms which can generate excess electrons, so Ce^4+^can be substituted by W^6+^and produce one or two excess electrons, and the excess electrons will be compensated by producing one or two Ce^3+^; these Ce^3+^ ions play a crucial role in the generation of oxygen vacancy due to its charge imbalance and unsaturated chemisorption bond; thus, it further enhances the catalytic activity greatly (Liu et al., 2020).

As shown in Figure 8D, O 1s peaks are fitted into two peaks at 528.0–530.0 and 530.0–532.0eV (according to Gaussian bands), and those peaks are ascribed to lattice oxygen (O^2−^, denoted as O_β_) and chemically adsorbed oxygen (O_2_^−^ and/or O^−^, denoted as O_α_), respectively (Zhang S. et al., 2015). Generally speaking, O_α_ is active oxygen species that has higher mobility than lattice oxygen, and it is the determining factor for low-temperature NH_3_-SCR reaction and soot oxidation. Because gas-phase NO is more easily obtained and reacts with active oxygen species and forms NO_2_, NO_2_ will react with NO and NH_3_ in the fast-SCR mode and react with soot directly. Hence, the rates for NH_3_-SCR and soot oxidation reactions were enhanced. Based on the results in Table 3 and Figure 1, the O_α_/O_β_ ratio of monometallic oxide Mn_2_O_3_, CeO_2_, and WO_3_ is lower than that of other as-prepared catalysts, which manifest the effect of supports and other doping metals. W_1_CeMnO_δ_/3DOM ZrTiO_4_ has the highest O_α_/O_β_ ratio of 51.5% among the as-prepared catalysts and shows the highest NO conversion rate at temperature T_m_. Therefore, the high ratio of O_α_/O_β_ plays an important role in the simultaneous catalytic elimination of PM and NO_x_.

### 3.6 H_2_-TPR

**FIGURE 9:**
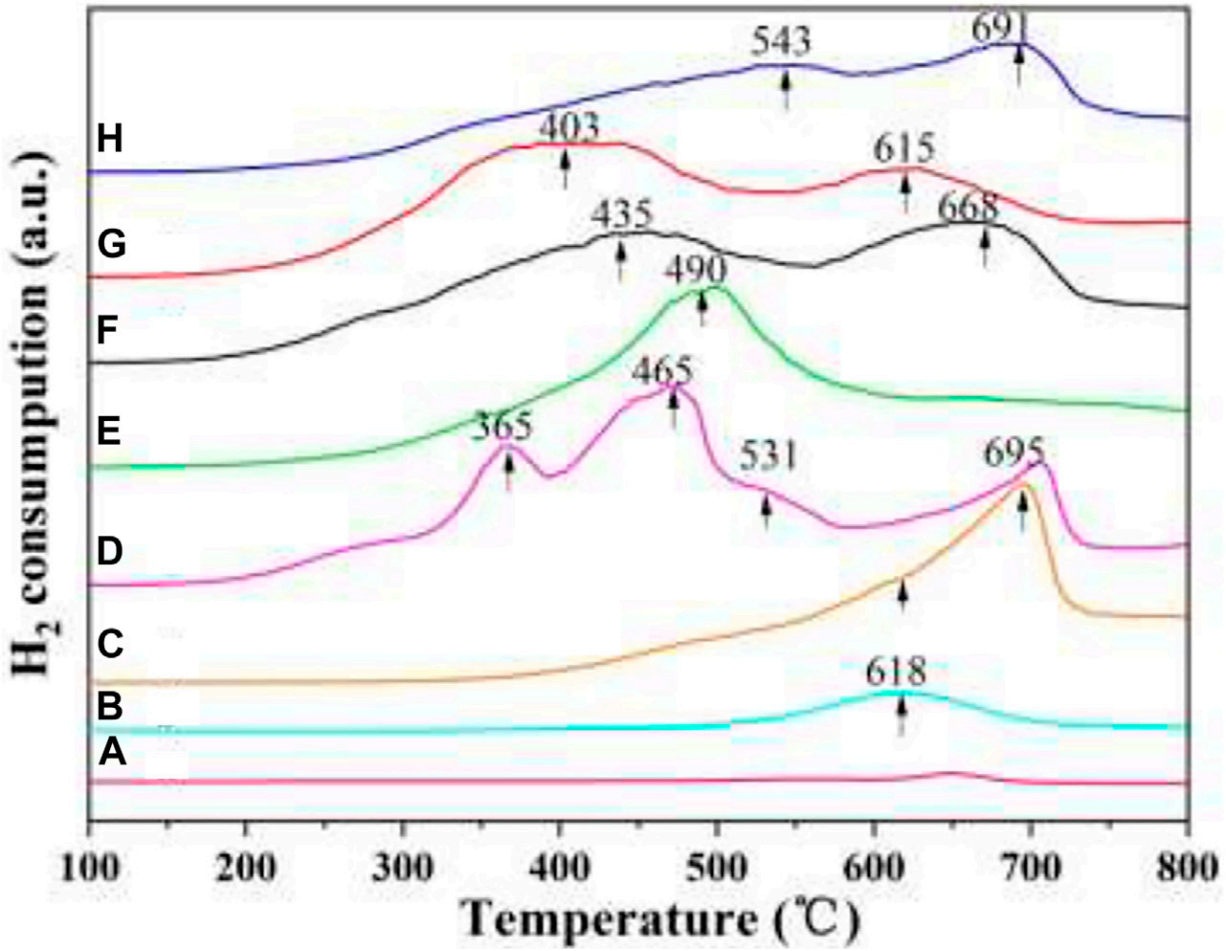
H_2_-TPR profiles of the 3DOM catalysts. **(A)** ZrTiO_4_; **(B)** CeO_2_/ZrTiO_4_; **(C)** W_1_CeO_δ_/ZrTiO_4_; **(D)** W_1_MnO_δ_/ZrTiO_4_; **(E)** W_1_CeMnO_δ_/TiO_2_; **(F)** W_0.5_CeMnO_δ_/ZrTiO_4_; **(G)** W_1_CeMnO_δ_/ZrTiO_4_; **(H)** W_2_CeMnO_δ_/ZrTiO_4_.

Catalysts with excellent redox properties are required in the simultaneous removal reaction. H_2_-TPR is usually applied for measuring the redox ability of catalysts. Figure 9 gives the H_2_-TPR results of the as-prepared catalysts. As shown in Figure 9A, the 3DOM ZrTiO_4_ support has almost no reduction peak, indicating that the redox capacity of the support is very weak. CeO_2_/3DOM ZrTiO_4_ has one reduction peak at 618°C, which is related to the reduction of surface oxygen species of ceria (Ma et al., 2012), since the reduction of bulk ceria occurred only above 750°C (Andreeva et al., 2004; Ndifor et al., 2007). When W is added into CeO_2_/3DOM ZrTiO_4_, the shoulder peak at 618°C belongs to the reduction of surface CeO_2_, and the peaks at 695°C are assigned to the reduction of surface WO_x_ (Ma et al., 2012). When Mn is doped into CeO_2_/ZrTiO_4_, three reduction peaks can be obtained. The first peak at 365°C is associated with the reduction of MnO_2_ to Mn_2_O_3_, the peak at 465°C is related to the reduction of surface Mn_2_O_3_ to Mn_3_O_4_, and the third shoulder peak at 531°C belongs to the reduction of surface Mn_3_O_4_ to MnO (Yu et al., 2014).

As shown in Figure 9E, W_1_CeMnO_δ_/3DOM TiO_2_ displays one reduction peak at 490 °C, which belongs to the overlapped reduction of surface oxygen. For W_x_CeMnO_δ_/3DOM ZrTiO_4_ catalysts, the TPR curves display two broad peaks, the former can belong to the reductions of the MnO_2_ to Mn_2_O_3_ and Mn_2_O_3_ to Mn_3_O_4_, and the latter may be related to the reductions of Mn_3_O_4_ to MnO, CeO_2_ to Ce_2_O_3_, and WO_x_ to W. Among the W_x_CeMnO_δ_/3DOM ZrTiO_4_ catalysts, the peaks of the W_1_CeMnO_δ_/3DOM ZrTiO_4_ catalyst shift to lower temperatures and the peak in the lower temperature has a wider reduction peak area, indicating that the 3DOM W_1_CeMnO_δ_/3DOM ZrTiO_4_ catalyst has strong reduction ability and much Mn^4+^ species. The Mn^4+^/Mn^3+^ ionic couple have proper redox characteristics and easily deliver more active sites for improving activity; therefore, the W_1_CeMnO_δ_/3DOM ZrTiO_4_ catalyst shows good simultaneous removal activity.

### 3.7 NH_3_-TPD

**FIGURE 10:**
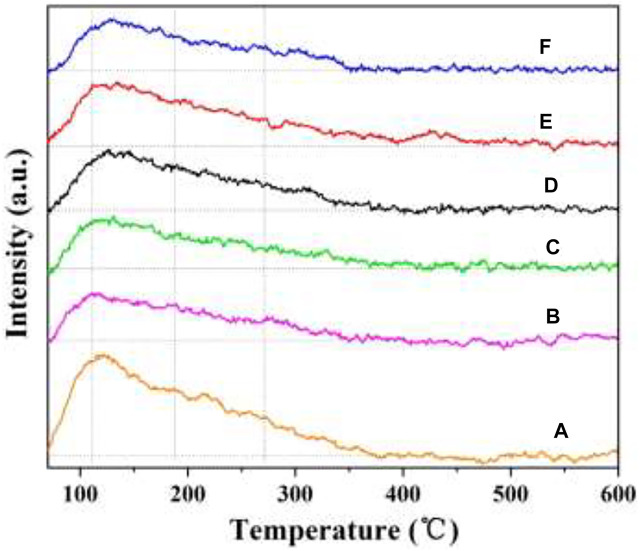
NH_3_-TPD profiles of the 3DOM catalysts. **(A)** W_1_CeO_δ_/ZrTiO_4_; **(B)** W_1_MnO_δ_/ZrTiO_4_; **(C)** W_1_CeMnO_δ_/TiO_2_; **(D)** W_0.5_CeMnO_δ_/ZrTiO_4_; **(E)** W_1_CeMnO_δ_/ZrTiO_4_; **(F)** W_2_CeMnO_δ_/ZrTiO_4_.

Surface acidity plays a significant role in NH_3_-SCR reaction (Xu et al., 2016; Xu et al., 2017). NH_3_-TPD was used to evaluate the amount and strength of surface acid sites. Therefore, the NH_3_-TPD profiles of the as-prepared catalysts were tested, and the results are shown in Figure 10. All the catalysts have broad peaks in the temperature range of 50–350°C, which is related to the desorption of NH_3_ on the weak and medium acid sites. Interestingly, these peaks are nearly at the same location, and no significant difference can be observed. Compared with the W_1_CeMnO_δ_/3DOM TiO_2_ catalyst, the peak area of the W_1_CeMnO_δ_/3DOM ZrTiO_4_ catalyst is larger, which indicates that the W_1_CeMnO_δ_/3DOM ZrTiO_4_ catalyst has more acid sites due to the doping of Zr in the ZrTiO_4_ support. In comparison with W_1_MnO_δ_/3DOM ZrTiO_4_ catalyst, more acid sites can be seen in W_1_CeMnO_δ_/3DOM ZrTiO_4_, and these acid sites may come from the electron-unsaturated W^6+^, as W^6+^enter the CeO_2_ lattice, leading to lower coordination between W^6+^ and surrounding oxygen, so it needs to share more electrons to balance the charge transfer. Therefore, the above analyses verify the experimental results that the W_1_CeMnO_δ_/3DOM ZrTiO_4_ catalyst has the highest NO removal activity.

## 4 Discussions

### 4.1 Effects of 3DOM Structure on Catalytic Performances

Soot combustion is the reaction of gas–solid–solid, and the contact efficiency between catalyst and soot is a significant factor for controlling the catalytic activity. 3DOM catalysts have highly ordered macroporous structures with diameter higher than 100 nm (SEM results). Because the diameter of soot particles (≈25 nm) is smaller than that of macropores, the large pores can capture soot particles and transfer soot particles to the inner active sites so that the active sites of catalysts can be fully utilized. Meanwhile, the diffusion resistance is reduced due to highly ordered macropores. Some previous work also confirmed the effect of the macroporous structure on improving catalytic activity (Yu et al., 2019; Yu X. et al., 2021).

In addition, the effects of NPs also play important roles in removing soot particles, such as the quantum size effects and surface effects. In this work, the active components are formed nanoparticles on the surface of 3DOM ZrTiO_4_ support. From the TEM results, it can be found that the diameter of active components is well falling into the scale of NPs. At the same time, gaseous reactants are more easily absorbed due to a lot of NPs on the surface of 3DOM ZrTiO_4_ supports, thus improving the efficiency of the catalytic reaction.

### 4.2 Possible Reaction Mechanism for Simultaneous deSoot an deNO_x_

**SCHEME 1:**
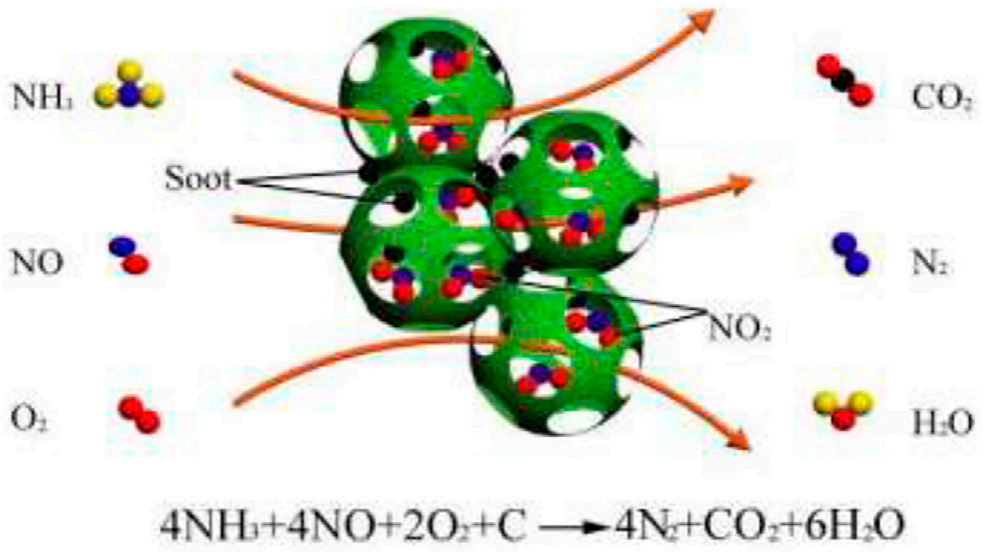
Possible reaction mechanisms for the simultaneous catalytic elimination of soot and NO_x_

To more deeply understand the reaction essence of simultaneous deSoot and deNO_x_, according to the results in this work and previous reports, the possible reaction mechanisms are also speculated and described in Scheme 1.

For the reaction of deSoot, based on the results of XPS (Figure 8) and H_2_-TPR (Figure 9), the 3DOM W_1_CeMnO_δ_/3DOM ZrTiO_4_ catalyst has more Mn^4+^, Ce^3+^, and O_α_ than other as-prepared catalysts, which indicates that it has more active sites, so gas-phase O_2_ molecules are more easily to be adsorbed and activated on the active sites (oxygen vacancies). On the one hand, adsorbed O_2_ forms active oxygen species, then soot traps the active oxygen species and forms surface oxygen–carbon complexes (SOC), and finally, the SOC further decomposes and produces CO_2_ and CO. On the other hand, these active oxygen species react with NO to form NO_2_; NO_2_ has stronger oxidation capacity than active oxygen species, so it can react with soot directly and changes the reaction path from gas–solid–solid to gas–gas–solid, thus accelerating the process of soot combustion. Therefore, the W_1_CeMnO_δ_/3DOM ZrTiO_4_ catalyst has a lower T_m_ value of 474°C **(** Figure 1).

**FIGURE 11:**
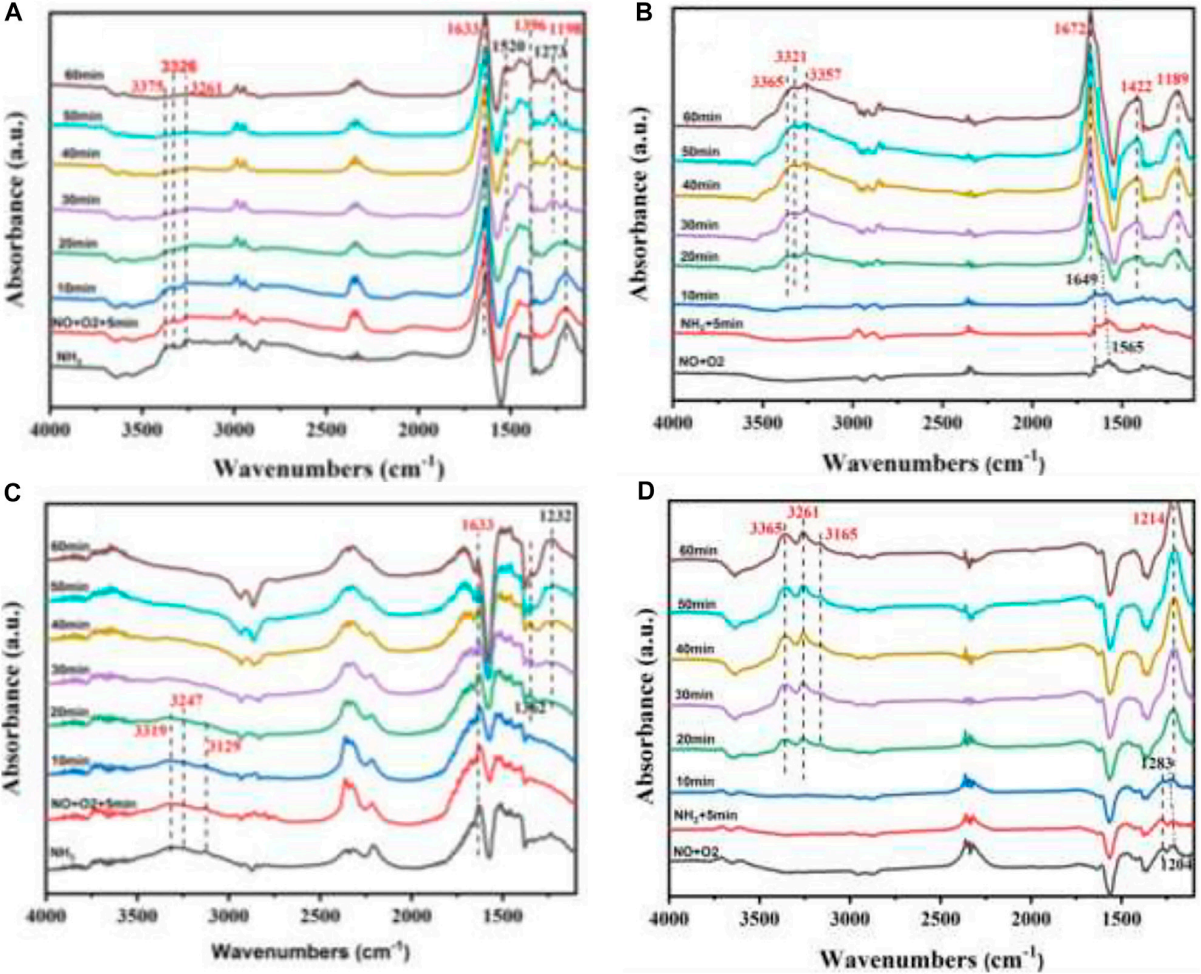
*In situ* DRIFTS spectra of the W_1_CeMnO_δ_/ZrTiO_4_ catalyst under different temperatures and reaction gases. **(A)** The reaction between NO + O_2_ and pre-adsorbed NH_3_ at 200°C; **(B)** reaction between NH_3_ and pre-adsorbed NO + O_2_ at 200°C; **(C)** reaction between NO + O_2_ and pre-adsorbed NH_3_ at 300°C; **(D)** reaction between NH_3_ and pre-adsorbed NO + O_2_ at 300°C.

For the reaction of deNO_x_, in order to investigate the SCR reaction mechanism of the W_1_CeMnO_δ_/3DOM ZrTiO_4_ catalyst, *in situ* DRIFTS were measured at 200 and 300°C, and the results are shown in Figure 11. As shown in Figure 11A, after being purged by N_2_, several absorbance bands were observed. The bands centered at 1,198 and 1,633 cm ^−1^ are ascribed to adsorbed NH_3_ on Lewis acidic sites, and the bands at 1,396 cm^−1^ belong to the coordinated NH^4+^on Bronsted acid sites, while the bands at 3,100–3,500 cm^−1^ are attributed to N-H stretching vibration of coordinated ammonia (Tan et al., 2018; Liu et al., 2020). When NO + O_2_ was introduced into the *in situ* reaction cell, all the bands belonging to the ammonia species are reduced gradually in their intensities with increasing time, and the IR bands (1,198 and 3,100–3,500 cm^−1^) disappear after 20 min. At the same time, the nitrate species including bidentate nitrate (1,273 cm^−1^) and monodentate nitrate (1,520 cm^−1^) begin to appear on the surface of the catalyst, including bidentate nitrate (1,273 cm^−1^) and monodentate nitrate (1,520 cm^−1^). These results indicate that the gas phase NO reacts with the coordinated NH_3_ on the surface of the W_1_CeMnO_δ_/3DOM ZrTiO_4_ catalyst through the E-R mechanism.

As shown in Figure 11B, after being purged by N_2_, bidentate nitrates (1,565 cm^−1^) and bridging nitrates (1,649 cm^−1^) (Liu et al., 2020; Wang et al., 2020) were formed on the surface of the W_1_CeMnO_δ_/3DOM ZrTiO_4_ catalyst because the mixture of NO + O_2_ was adsorbed on the surface of the catalyst. When NH_3_ was introduced into the *in situ* reaction cell, the bands related to nitrate species decreased gradually in the first 10 min, and no adsorption peaks were found for NH_3_ species, indicating that the gas phase NH_3_ reacts with the nitrate species. After 20 min, the bands belonging to NH_3_ species begin to appear, indicating the existence of ammonia on the surface of the catalyst; the bands at 1,189 and 1,672 cm ^−1^ belong to the adsorbed NH_3_ on Lewis acidic sites; the bands at 1,422 cm^−1^ belong to the coordinated NH^4+^on Bronsted acid sites; and the peaks at 3,100–3,500 cm^−1^ belong to N-H stretching vibration of coordinated ammonia. Interestingly, the bands at 1,565cm^−1^ (bidentate nitrates) begin to shift after 5 min and decrease slowly within 15 min. After 15 min, the peak at 1,565 cm^−1^ (bidentate nitrates) disappeared and the peak belonged to the NH_3_ species increased gradually, so the IR band at 1,672 cm^−1^ may be caused by the overlap of the bidentate nitrates (1,565 cm^−1^), bridging nitrates (1,649 cm^−1^), and the coordinated NH_3_ adsorbed on the Lewis acid site (1,672 cm^−1^). Based on the above results and discussion, it can be found that bidentate nitrate (1,565 cm^−1^) and active NH_3_ can coexist on the surface of the W_1_CeMnO_δ_/3DOM ZrTiO_4_ catalyst and the SCR reaction mechanism of the W_1_CeMnO_δ_/3DOM ZrTiO_4_ catalyst can be both E-R mechanism and L-H mechanism at 200°C.

Figures 11C, D show the *in situ* DRIFT spectra of the W_1_CeMnO_δ_/3DOM ZrTiO_4_ catalyst measured at 300°C. As shown in Figure 11C, similarly, the catalyst was first treated by NH_3_. After being purged by N_2_, the bands centered at 1,633 cm ^−1^ are ascribed to the adsorbed NH_3_ on Lewis acidic sites, and the bands at 3,100–3,500 cm^−1^ are attributed to N-H stretching vibration of coordinated ammonia. When NO + O_2_ was introduced into the *in situ* reaction cell, the intensities of all the bands belonging to the ammonia species gradually reduced, and the IR bands (3,100–3,500 cm^−1^) disappeared after 20 min. The bands, which belong to nitrate species, appear; the band centered at 1,232 cm ^−1^ is ascribed to bridging nitrate; and the band at 1,362 cm^−1^ is attributed to bidentate nitrate. These results indicate that gas phase NO reacts with coordinated NH_3_ also through the E-R mechanism at 300°C.

As shown in Figure 11D, the catalyst was first treated with NO + O_2_. After purging with N_2_, the bands assigned to bridging nitrates (1,204 cm^−1^) and monodentate nitrates (1,283 cm^−1^) were detected to demonstrate the formation of these nitrate species on the surface of the catalyst. When NH_3_ was introduced, the band intensity related to nitrate species decreased gradually in the first 10 min, and no adsorption peaks were found for NH_3_ species. After 20 min, several bands began to appear; the bands at 1,214 cm^−1^ are ascribed to adsorbed NH_3_ on Lewis acidic sites, and the bands at 3,100–3,500 cm^−1^ are attributed to N-H stretching vibrational of coordinated ammonia. Similar to the case at 200°C, the bands at 1,204 cm^−1^ were shifted after 5 min and band intensity decreased slowly. After 15 min, the peaks at 1,204 and 1,283 cm^−1^ disappeared, and the intensity of the peak belonging to the NH_3_ species was increased gradually. The IR band at 1,214 cm^−1^ may be the overlap of the bridging nitrates (1,204 cm^−1^), monodentate nitrates (1,283 cm^−1^), and the adsorbed NH_3_ species. These results indicate that the reaction at 300°C is similar to the reaction at 200°C.

To sum up, the reaction mechanism for the simultaneous deSoot and deNO_x_ is a complex mechanism that mixes two reactions together. In this work, four mechanisms, including the active oxygen mechanism, NO_2_-assisted mechanism, L-H mechanism, and E-R mechanism, were proposed, and these four mechanisms work together in the simultaneous elimination reaction.

## 5 Conclusion

3DOM ZrTiO_4_ support and a series of W_x_CeMnO_δ_/3DOM ZrTiO_4_ oxide catalysts were fabricated by the colloidal crystal template method and applied to the simultaneous elimination of PM and NO_x_. Based on the analyses of characterization and activity evaluation results, the as-prepared catalysts have a high-quality 3DOM structure, and the W_1_CeMnO_δ_/3DOM ZrTiO_4_ catalyst exhibits the best catalytic performance due to the perfect structure, large surface area, abundant acid sites, and the synergistic effect among the active components. Among the as-prepared catalysts, W_1_CeMnO_δ_/3DOM ZrTiO_4_ exhibits the widest temperature window (250–396°C) at a lower temperature for 90% NO conversion but also has the highest NO conversion rate (52%) at the temperature of T_m_ for soot combustion. The catalytic mechanism for the simultaneous elimination of soot particulate matter and nitrogen oxides over W_1_CeMnO_δ_/3DOM ZrTiO_4_ catalyst is mainly governed *via* the active oxygen mechanism, NO_2_-assisted mechanism, L-H mechanism, and E-R mechanism. The as-prepared W_x_CeMnO_δ_/3DOM ZrTiO_4_ catalysts have application prospects for the simultaneous elimination of soot particulate matter and nitrogen oxides from diesel engine exhausts, owing to easy preparation, low cost, and high catalytic activity.

## Data Availability

The original contributions presented in the study are included in the article/Supplementary Material, further inquiries can be directed to the corresponding authors.
